# The Nuclear Export Signal of IκBα Drives RelB Oscillations in the Noncanonical NF‐κB Pathway

**DOI:** 10.1111/gtc.70135

**Published:** 2026-07-02

**Authors:** Takao Seki, Shelly Davis, Shigeki Miyamoto, Taishin Akiyama, Hiroyasu Nakano, Jun‐ichiro Inoue, Katsuhide Okunishi

**Affiliations:** ^1^ Division of Cellular and Molecular Biology, Department of Cancer Biology The Institute of Medical Science, the University of Tokyo Tokyo Japan; ^2^ Laboratory for Immune Homeostasis RIKEN Center for Integrative Medical Sciences Yokohama Japan; ^3^ Department of Biochemistry, Faculty of Medicine Toho University Tokyo Japan; ^4^ Department of Oncology, University of Wisconsin Carbone Cancer Center University of Wisconsin Madison Wisconsin USA; ^5^ Unit of Host Defense, Faculty of Medicine Toho University Tokyo Japan; ^6^ Research Administration Organization Toho University Tokyo Japan

## Abstract

The noncanonical NF‐κB pathway regulates immune development and inflammation through RelB nuclear translocation, yet the dynamics of this process at the single‐cell level remain poorly understood. Using live‐cell imaging of RelB‐Venus knock‐in mouse embryonic fibroblasts, we show that LTβR or TWEAK stimulation induces four distinct RelB nuclear translocation patterns: oscillating, prolonged activation, transient activation, and non‐responding. Approximately 40% of cells exhibited oscillatory behavior with a predominant period of 1.5–2.0 h, similar to RelA oscillations in the canonical pathway. Mechanistically, RelB oscillations required CRM1‐mediated nuclear export, ongoing protein synthesis, NIK‐dependent noncanonical signaling, and the IκBα nuclear export signal. Knockdown or knockout of *Nfkb2* (encoding p100) induced spontaneous RelB oscillations without stimulation, identifying p100 as a threshold regulator that controls oscillation probability through cytoplasmic sequestration of RelB. Co‐immunoprecipitation analysis revealed dynamic RelB‐IκBα complex formation in both cytoplasmic and nuclear fractions following LTβR stimulation. Furthermore, disruption of RelB oscillations in *Nfkbia* ^*NES/NES*^ cells was associated with impaired induction of NF‐κB target genes. These findings provide the first experimental characterization of RelB oscillatory dynamics and reveal both conserved and pathway‐specific mechanisms governing noncanonical NF‐κB signaling.

## Graphical abstract

The IκBα nuclear export signal drives oscillatory dynamics of RelB in the noncanonical NF‐κB pathway. Following LTβR stimulation, p100 processing releases RelB for nuclear import, while IκBα‐mediated CRM1‐dependent nuclear export enables repeated RelB oscillations, regulating NF‐κB target gene expression.

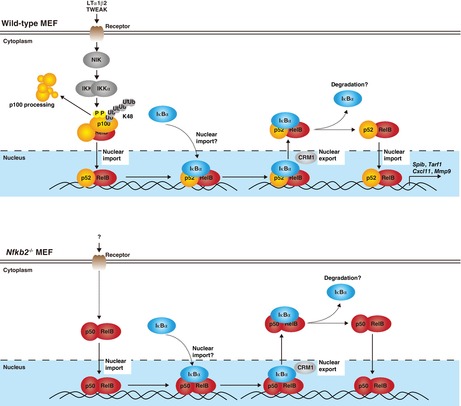

## 1 Introduction

The nuclear factor‐κB (NF‐κB) family of transcription factors plays essential roles in regulating immune responses, inflammation, cell survival, and development (Hayden and Ghosh 2012; Zhang et al. 2017). In mammals, the NF‐κB family comprises five members, RelA, RelB, c‐Rel, p50, and p52, which form various homo‐ and heterodimers to regulate distinct sets of target genes. NF‐κB activity is tightly controlled through two major signaling pathways, the canonical and noncanonical pathways, each with distinct activation mechanisms and biological functions.

The canonical NF‐κB pathway is primarily mediated by RelA:p50 heterodimers and responds rapidly to diverse stimuli, including cytokines, pathogen‐associated molecular patterns, and cellular stress. A landmark discovery in NF‐κB signaling was the observation that RelA exhibits oscillatory nuclear‐cytoplasmic translocation following TNF stimulation (Hoffmann et al. 2002; Nelson et al. 2004). These oscillations are driven by negative feedback through newly synthesized IκBα, which exports RelA from the nucleus via its nuclear export signal (NES), and occur with a period of approximately 1.5–2 h. They have been shown to encode information about stimulus strength and duration (Ashall et al. 2009; Tay et al. 2010). Computational modeling and experimental studies have revealed that RelA oscillations enable cells to discriminate between transient and sustained inflammatory signals, leading to distinct gene expression programs (Sung et al. 2009; Werner et al. 2005). These findings establish oscillatory dynamics as a fundamental mechanism for information processing in the canonical NF‐κB pathway.

The noncanonical NF‐κB pathway is activated by a specific subset of receptors, including lymphotoxin β receptor (LTβR), BAFF receptor, CD40, and TWEAK receptor (Fn14), and primarily signals through RelB:p52 heterodimers (Sun 2017). This pathway plays crucial roles in lymphoid organogenesis, B cell maturation, and adaptive immunity. Activation requires the accumulation of NF‐κB‐inducing kinase (NIK) and subsequent IKKα‐mediated phosphorylation and processing of p100 to p52, which releases RelB from cytoplasmic sequestration (Senftleben et al. 2001; Xiao et al. 2001). The noncanonical pathway is thus characterized by slow kinetics compared to the canonical pathway. While the molecular components of the noncanonical pathway have been well characterized, the dynamic behavior of RelB nuclear translocation has remained unexplored.

Whether RelB exhibits oscillatory behavior similar to RelA has not been investigated. This knowledge gap is significant because oscillatory dynamics have emerged as a general principle of cellular signaling, enabling temporal information encoding and frequency‐dependent gene regulation (Levine et al. 2013; Purvis and Lahav 2013). The noncanonical pathway controls biological processes distinct from those regulated by the canonical pathway, raising the question of whether different NF‐κB pathways employ similar temporal coding mechanisms.

To address these questions, we previously generated RelB‐Venus knock‐in mice, enabling visualization of endogenous RelB dynamics in dendritic cells (Seki et al. 2015). Building upon this tool, we now report the first experimental characterization of RelB nuclear translocation dynamics following noncanonical NF‐κB pathway activation. Using live‐cell imaging of mouse embryonic fibroblasts (MEFs) derived from RelB‐Venus knock‐in mice, we demonstrate that noncanonical NF‐κB pathway activation induces RelB oscillations with periods similar to RelA, but with pronounced cell‐to‐cell heterogeneity. We further reveal that RelB oscillations require IκBα‐dependent nuclear export and continuous protein synthesis, while p100 functions as a threshold regulator controlling the probability of oscillatory behavior. Disruption of RelB oscillations through mutation of the IκBα NES alters the expression of immune response genes, indicating the functional significance of these dynamics. Our findings establish oscillatory dynamics as a conserved feature across both canonical and noncanonical NF‐κB pathways while highlighting pathway‐specific regulatory mechanisms.

## 2 Results

### 2.1 Noncanonical NF‐κB Pathway Activation Induces Diverse RelB Nuclear Translocation Dynamics

**FIGURE 1:**
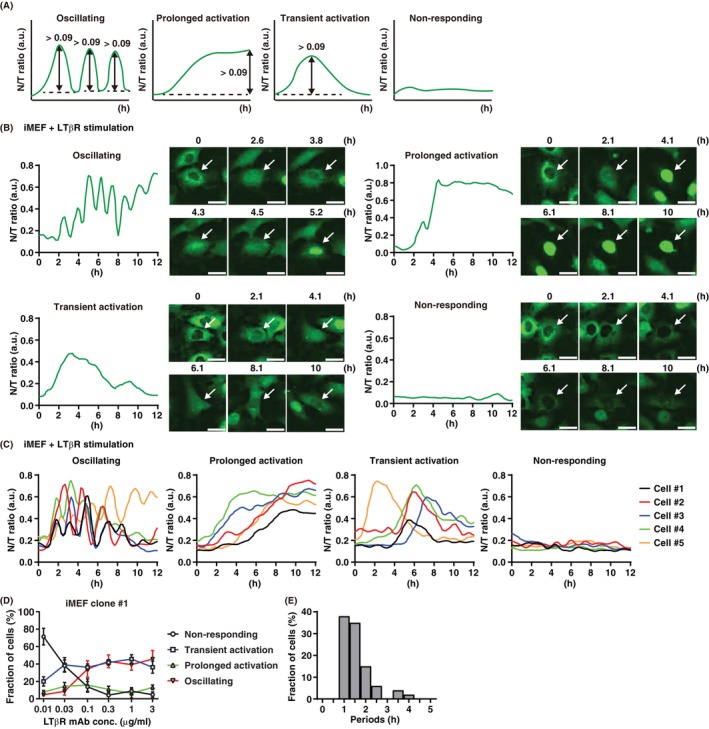
Noncanonical NF‐κB pathway activation induces diverse RelB nuclear translocation dynamics. All experiments were performed using immortalized MEFs (iMEFs) established from RelB‐Venus knock‐in mice. (A) Schematic representation of four distinct RelB nuclear translocation patterns (N/T ratio > 0.09 defined as nuclear translocation). Oscillating, repetitive nuclear translocation cycles; prolonged activation, sustained nuclear accumulation; transient activation, single peak followed by return to baseline; non‐responding, no nuclear translocation. (B) Representative time‐lapse images and quantification of RelB‐Venus nuclear translocation in iMEFs stimulated with anti‐LTβR mAb (1 μg/mL). Arrows indicate the tracked cell. Scale bar: 10 μm. (C) Single‐cell N/T ratio traces for each response category in RelB‐Venus iMEFs stimulated with anti‐LTβR mAb (1 μg/mL). Five representative cells per category are shown. (D) Fraction of iMEFs (clone #1) displaying each response pattern at the indicated anti‐LTβR mAb concentrations. (E) Distribution of oscillation periods in oscillating iMEFs determined by FFT analysis (*n* = 48 cells). All experiments are representative of at least three independent experiments.

To examine RelB subcellular dynamics following noncanonical NF‐κB pathway activation, we stimulated primary MEFs (pMEFs) derived from RelB‐Venus knock‐in mice with an agonistic anti‐LTβR monoclonal antibody (LTβR mAb) and monitored RelB localization by time‐lapse fluorescence microscopy (Seki et al. 2015). Based on the nuclear‐to‐total fluorescence ratio (N/T ratio), we classified single‐cell responses into four categories: “oscillating” (repetitive nuclear‐cytoplasmic cycling), “prolonged activation” (sustained nuclear accumulation), “transient activation” (a single peak returning to baseline), and “non‐responding” (no nuclear accumulation) (Figures 1A and S1).

To enable more systematic analysis, we established immortalized MEF cell lines (iMEFs) from RelB‐Venus knock‐in mice. iMEFs displayed the same four response categories (Figure 1B,C), and the fraction of oscillating cells increased in a concentration‐dependent manner with LTβR mAb, reaching approximately 40% at the highest dose tested (Figure 1D). Multiple independent iMEF clones showed similar dose‐dependent responses (Figure S1). Stimulation with TWEAK, another noncanonical NF‐κB pathway ligand, likewise induced oscillatory RelB dynamics in a dose‐dependent manner (Figure S1). Fast Fourier Transform (FFT) of both pMEFs and iMEFs revealed a predominant oscillation period of 1.5–2.0 h regardless of the ligands used (Figures 1E and S1), which is comparable to the oscillation period reported for RelA in the canonical NF‐κB pathway.

### 2.2 RelB Oscillations Require CRM1‐Mediated Nuclear Export and Protein Synthesis

RelA oscillations in the canonical NF‐κB pathway depend on newly synthesized IκBα, which drives nuclear export of RelA via its NES (Nelson et al. 2004). We hypothesized that RelB oscillations might similarly require CRM1‐mediated nuclear export and de novo protein synthesis.

**FIGURE 2:**
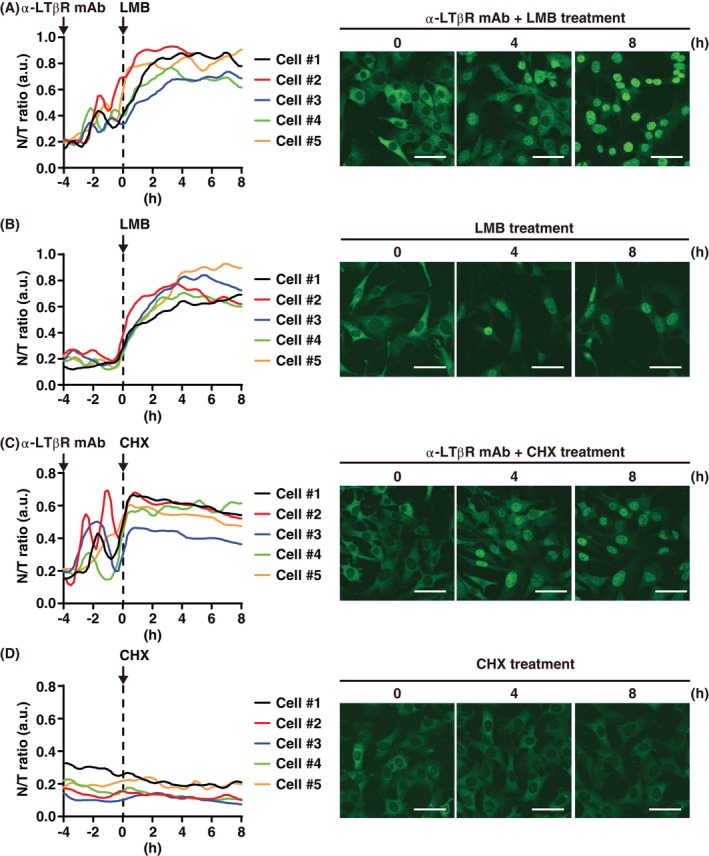
RelB oscillations require CRM1‐mediated nuclear export and protein synthesis. All experiments were performed using iMEFs established from RelB‐Venus knock‐in mice. (A) iMEFs were stimulated with anti‐LTβR mAb (1 μg/mL) for 4 h, followed by addition of LMB (1 ng/mL) at time 0. Single‐cell N/T ratio traces and representative time‐lapse images are shown. Scale bar, 50 μm. (B) LMB (1 ng/mL) was added to unstimulated iMEFs at time 0. Single‐cell N/T ratio traces and representative time‐lapse images are shown. Scale bar, 50 μm. (C) iMEFs were stimulated with anti‐LTβR mAb (1 μg/mL) for 4 h, followed by addition of cycloheximide (CHX; 2.5 μg/mL) at time 0. Single‐cell N/T ratio traces and representative time‐lapse images are shown. Scale bar, 50 μm. (D) CHX (2.5 μg/mL) was added to unstimulated iMEFs at time 0. Single‐cell N/T ratio traces and representative time‐lapse images are shown. Scale bar, 50 μm. Time indicates hours before (negative values) and after (positive values) drug addition. All experiments are representative of at least three independent experiments.

To test this, we stimulated iMEFs with anti‐LTβR mAb for 4 h to establish oscillatory behavior, then added leptomycin B (LMB), a specific inhibitor of the nuclear export receptor CRM1. LMB treatment abolished oscillations and caused sustained nuclear retention of RelB (Figure 2A). Notably, LMB treatment alone, without prior LTβR stimulation, also induced nuclear accumulation of RelB (Figure 2B), indicating that RelB undergoes constitutive CRM1‐dependent nuclear export even in unstimulated cells.

We next applied the same experimental design using cycloheximide (CHX), a protein synthesis inhibitor. CHX likewise abolished RelB oscillations and resulted in nuclear retention (Figure 2C). However, CHX treatment alone did not induce nuclear accumulation in unstimulated cells (Figure 2D), in contrast to LMB. Together, these results demonstrate that RelB oscillations require both CRM1‐mediated nuclear export and ongoing protein synthesis. Furthermore, the nuclear accumulation of RelB following LMB treatment of unstimulated cells indicates that RelB undergoes constitutive nuclear import and CRM1‐dependent nuclear export in the absence of stimulation, reflecting a dynamic equilibrium between these two processes. This constitutive nucleocytoplasmic movement occurs independently of new protein synthesis, as CHX treatment alone did not induce nuclear accumulation in unstimulated cells.

### 2.3 RelB Oscillations Require the Noncanonical but Not Canonical NF‐κB Pathway

To determine whether the canonical NF‐κB pathway contributes to RelB oscillations, we examined *Ikbkg* ^*−/−*^ iMEFs lacking NEMO (IKKγ), an essential component of the canonical pathway. Western blot analysis confirmed that *Ikbkg* ^*−/−*^ iMEFs failed to degrade IκBα in response to TNF, while LTβR‐induced p100 processing to p52 remained intact (Figure S2A). When *Ikbkg* ^*−/−*^ iMEFs were transduced with a RelB‐Venus lentiviral vector and stimulated with anti‐LTβR mAb, RelB oscillations were indistinguishable from those in *Ikbkg* ^*+/+*^ iMEFs (Figure S2B,C), demonstrating that the canonical pathway is dispensable for RelB oscillations.

We next asked whether NIK (NF‐κB‐inducing kinase, encoded by *Map3k14*), the key upstream kinase of the noncanonical pathway, is required for RelB oscillations. Knockdown of *Map3k14* by siRNA substantially reduced NIK protein levels (Figure S2D) and blocked LTβR‐induced p100 processing to p52, while TNF‐induced IκBα degradation remained unaffected (Figure S2E). Under these conditions, LTβR stimulation failed to induce RelB oscillations, and the majority of cells showed no nuclear translocation of RelB (Figure S2F). Together, these results demonstrate that NIK‐dependent noncanonical NF‐κB signaling is required for RelB oscillations, whereas the canonical pathway is not.

### 2.4 p100 Controls RelB Oscillation Threshold by Cytoplasmic Sequestration

**FIGURE 3:**
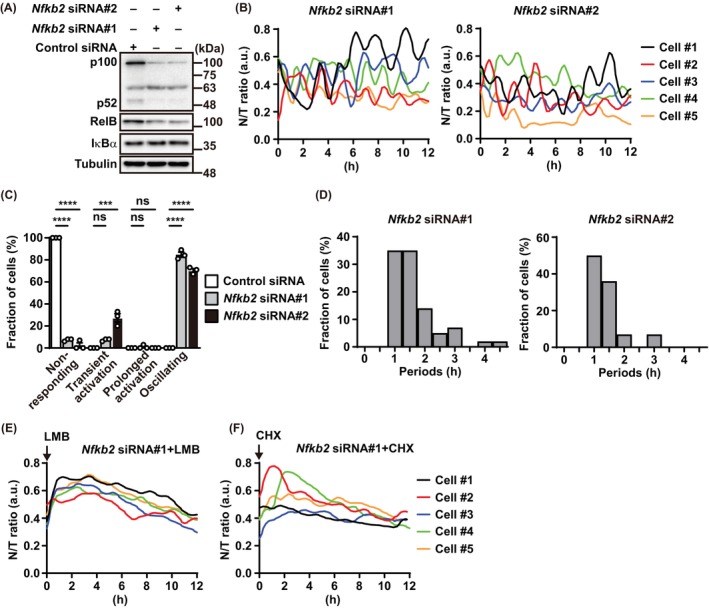
p100 controls RelB oscillation by cytoplasmic sequestration. All experiments were performed using iMEFs established from RelB‐Venus knock‐in mice. (A) Western blot analysis of p100, p52, RelB, IκBα, and Tubulin in iMEFs transfected with control or *Nfkb2* siRNA (#1 and #2). (B) Single‐cell N/T ratio traces in iMEFs transfected with *Nfkb2* siRNA #1 (left) or #2 (right) without LTβR stimulation. Five representative cells per condition are shown. (C) Fraction of iMEFs displaying each response pattern following *Nfkb2* siRNA transfection. (D) Distribution of oscillation periods in oscillating *Nfkb2* knockdown iMEFs determined by FFT analysis. (E) Single‐cell N/T ratio traces in *Nfkb2* siRNA#1‐transfected iMEFs treated with LMB (1 ng/mL) at time 0. Five representative cells are shown. (F) Single‐cell N/T ratio traces in *Nfkb2* siRNA#1‐transfected iMEFs treated with CHX (2.5 μg/mL) at time 0. Five representative cells are shown. Statistical significance was determined by one‐way ANOVA with Tukey's multiple comparisons test (C): ***p < 0.001; ****p < 0.0001; ns, not significant. All experiments are representative of at least two independent experiments.

p100 sequesters RelB in the cytoplasm via its C‐terminal nuclear export signal, and LTβR stimulation releases RelB by promoting p100 processing to p52 (Busino et al. 2012). To examine the role of p100 in RelB oscillations, we knocked down *Nfkb2* in iMEFs using two independent siRNAs, which efficiently reduced both p100 and p52 protein levels (Figure 3A). Strikingly, *Nfkb2* knockdown alone induced spontaneous RelB oscillations in more than 80% of cells without LTβR stimulation (Figure 3B,C), while the predominant oscillation period remained at 1.0–2.0 h (Figure 3D). Treatment of *Nfkb2* knockdown cells with LMB or CHX abolished these spontaneous oscillations (Figure 3E,F), indicating that CRM1‐mediated nuclear export and ongoing protein synthesis remain essential regardless of p100 levels.

To confirm these findings genetically, we generated *Nfkb2* ^*−/−*^ iMEFs by CRISPR‐Cas9 editing (Figure S3A,B). Consistent with the siRNA results, *Nfkb2* ^*−/−*^ cells exhibited spontaneous RelB oscillations without stimulation, with oscillation periods similar to those in knockdown cells (Figure S3C–E). Together, these results demonstrate that p100 sets the threshold for RelB oscillations through cytoplasmic sequestration, while the core oscillatory mechanism remains dependent on CRM1‐mediated nuclear export and protein synthesis.

### 2.5 RelB Oscillations Require IκBα Nuclear Export Signal

Since CRM1‐mediated nuclear export is essential for RelB oscillations, we hypothesized that IκBα, a known CRM1 cargo, might mediate RelB nuclear export and thereby contribute to RelB oscillatory dynamics. We first asked whether IκBα itself is required by generating *Nfkbia* ^*−/−*^ iMEFs using CRISPR‐Cas9 editing (Figure S4A,B). *Nfkbia* ^*−/−*^ iMEFs showed only a modest reduction in the proportion of oscillating cells compared to *Nfkbia* ^*+/+*^ iMEFs, but FFT analysis revealed that oscillation periods were longer in *Nfkbia* ^*−/−*^ iMEFs (Figure S4C–E), suggesting that IκBα contributes to efficient oscillations but is not strictly required.

**FIGURE 4:**
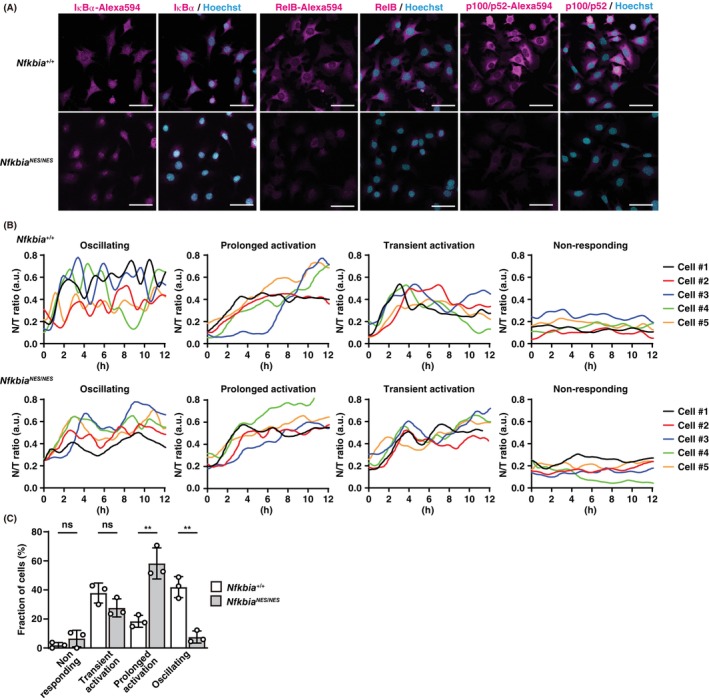
RelB oscillations require nuclear export signal in IκBα. Immunofluorescence experiments (A) were performed using *Nfkbia⁺/⁺* and *Nfkbia* ^*NES/NES*^ iMEFs. For live‐cell imaging (B and C), *Nfkbia* ^*NES/NES*^ iMEFs were transduced with the lentiviral RelB‐Venus vector prior to stimulation. (A) Immunofluorescence analysis of IκBα, RelB, and p100/p52 localization in *Nfkbia* ^*+/+*^ and *Nfkbia* ^*NES/NES*^ iMEFs at 6 h after anti‐LTβR mAb stimulation (1 μg/mL). Cells were stained with Alexa Fluor 594‐conjugated antibodies (magenta) and Hoechst 33342 (blue). Scale bars, 50 μm. (B) Single‐cell N/T ratio traces for each response category in *Nfkbia* ^*+/+*^ (upper panels) and *Nfkbia* ^*NES/NES*^ (lower panels) iMEFs following anti‐LTβR mAb stimulation (1 μg/mL). Five representative cells per category are shown. (C) Fraction of *Nfkbia* ^*+/+*^ and *Nfkbia* ^*NES/NES*^ iMEFs displaying each response pattern following anti‐LTβR mAb stimulation (1 μg/mL). Statistical significance was determined by two‐tailed unpaired Student's *t*‐test (C): **p < 0.01; ns, not significant. All experiments are representative of at least two independent experiments.

We next examined *Nfkbia* ^*NES/NES*^ iMEFs, in which the IκBα NES is replaced with alanine residues, causing constitutive nuclear retention of IκBα (Wuerzberger‐Davis et al. 2011). Immunofluorescence confirmed nuclear localization of IκBα in *Nfkbia* ^*NES/NES*^ iMEFs, in contrast to the cytoplasmic distribution in wild‐type cells (Figure 4A). *Nfkbia* ^*NES/NES*^ iMEFs also showed markedly reduced endogenous p100 and RelB protein levels (Figure 4A), consistent with previously reported effects of nuclear IκBα on basal NF‐κB target gene expression (Wuerzberger‐Davis et al. 2011). When *Nfkbia* ^*NES/NES*^ iMEFs were transduced with a RelB‐Venus lentiviral vector and stimulated with anti‐LTβR mAb, RelB oscillations were severely impaired, as shown by representative single‐cell N/T ratio traces (Figure 4B), with the proportion of oscillating cells falling below 10% compared to approximately 40% in *Nfkbia* ^*+/+*^ iMEFs, and a concomitant increase in prolonged activation (Figure 4C). Together, these results demonstrate that the IκBα NES is required for RelB oscillations, and that nuclear‐retained IκBα actively disrupts the oscillatory response in a manner that cannot be compensated by other mechanisms, in contrast to the partial compensation seen in *Nfkbia* ^*−/−*^ iMEFs.

### 2.6 Dynamic RelB‐IκBα Complex Formation Underlies RelB Oscillation Machinery

**FIGURE 5:**
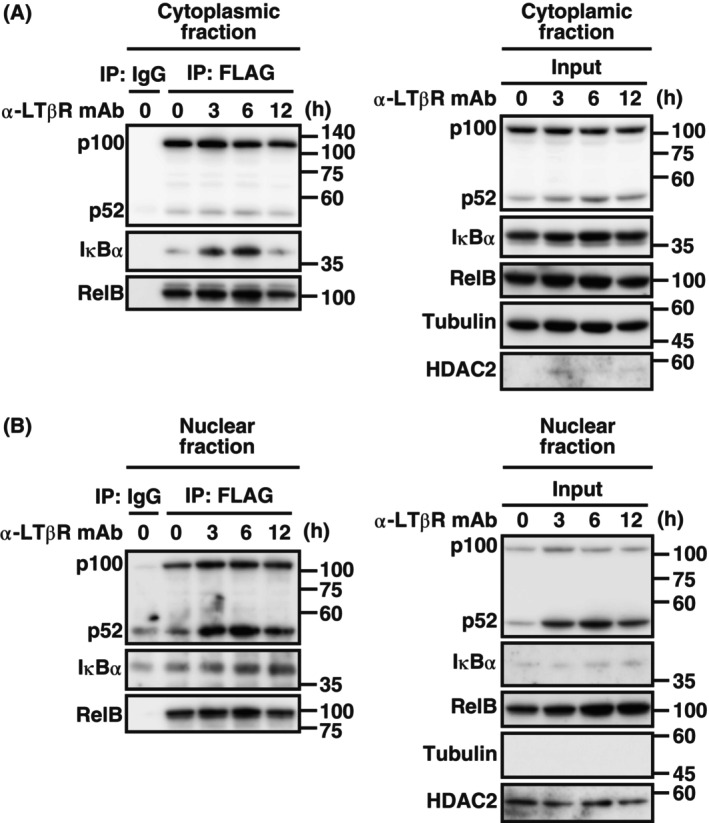
Dynamic RelB‐IκBα complex formation underlies noncanonical NF‐κB activation. All experiments were performed using iMEFs established from RelB‐Venus knock‐in mice. (A) Co‐immunoprecipitation of RelB‐Venus‐2 × FLAG from the cytoplasmic fraction of iMEFs stimulated with anti‐LTβR mAb (1 μg/mL) at the indicated time points. FLAG immunoprecipitates and input fractions were analyzed by western blot for p100, p52, IκBα, and RelB. Tubulin and HDAC2 serve as cytoplasmic and nuclear fraction markers, respectively. (B) Co‐immunoprecipitation of RelB‐Venus‐2 × FLAG from the nuclear fraction of iMEFs stimulated as in (A). All experiments are representative of at least two independent experiments.

To examine the molecular interactions underlying RelB oscillations, we performed co‐immunoprecipitation (co‐IP) experiments using iMEFs from RelB‐Venus‐2 × FLAG knock‐in mice stimulated with anti‐LTβR mAb. RelB formed complexes with p100, p52, and IκBα in both cytoplasmic and nuclear fractions (Figure 5A,B). The dynamics of RelB‐IκBα complex formation differed between compartments: cytoplasmic RelB‐IκBα complexes peaked at 3–6 h and declined by 12 h, whereas nuclear RelB‐IκBα complexes were weakly but reproducibly detected above the IgG control and accumulated progressively up to 12 h, consistent with a minor but functionally relevant pool of IκBα in the nuclear compartment.

To determine whether RelB‐IκBα interactions occur independently of p100, we performed the same co‐IP analysis in *Nfkb2* ^*−/−*^ iMEFs. In the absence of p100/p52, RelB formed complexes with p105/p50 and IκBα in both cytoplasmic and nuclear fractions (Figure S5A,B), demonstrating that p100 is not required for RelB‐IκBα interaction. These results indicate that IκBα‐mediated nuclear export of RelB operates independently of p100 and represents a core step in the oscillatory mechanism.

### 2.7 Disruption of RelB Oscillations Is Associated With Impaired NF‐κB Target Gene Expression

**FIGURE 6:**
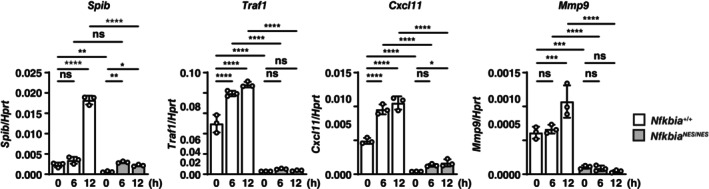
Disruption of RelB oscillations is associated with impaired NF‐κB target gene expression. Quantitative RT‐PCR analysis of *Spib*, *Traf1*, *Cxcl11*, and *Mmp9* expression in *Nfkbia* ^*+/+*^ and *Nfkbia* ^*NES/NES*^ iMEFs stimulated with anti‐LTβR mAb (1 μg/mL) for the indicated time points. Expression levels were normalized to *Hprt*. Results represent mean ± SD of triplicates from two independent experiments. Statistical significance was determined by two‐way ANOVA with Tukey's multiple comparisons test: *p < 0.05; **p < 0.01; ***p < 0.001; ****p < 0.0001; ns, not significant.

To examine whether disruption of RelB oscillatory dynamics is associated with changes in NF‐κB target gene expression, we measured expression of four NF‐κB target genes induced by LTβR stimulation (*Spib*, *Traf1*, *Cxcl11*, and *Mmp9*) by quantitative RT‐PCR in *Nfkbia* ^*+/+*^ and *Nfkbia* ^*NES/NES*^ iMEFs at 0, 6, and 12 h after anti‐LTβR mAb stimulation. All four genes were robustly induced in wild‐type cells, whereas *Nfkbia* ^*NES/NES*^ iMEFs showed markedly reduced induction as well as lower basal expression levels (Figure 6). However, since *Nfkbia* ^*NES/NES*^ iMEFs have impaired canonical NF‐κB signaling and reduced basal RelB and p100 expression (Wuerzberger‐Davis et al. 2011), and since LTβR stimulation may weakly activate the canonical pathway, the reduced gene expression in *Nfkbia* ^*NES/NES*^ iMEFs cannot be attributed solely to the disruption of RelB oscillations.

## 3 Discussion

In this study, we provide the first experimental characterization of oscillatory RelB dynamics in the noncanonical NF‐κB pathway. Using live‐cell imaging of RelB‐Venus knock‐in MEFs, we showed that LTβR or TWEAK stimulation induces diverse RelB nuclear translocation patterns, with approximately 40% of cells exhibiting oscillatory behavior. While a recent mathematical modeling study predicted RelB oscillations (Umegaki et al. 2024), our work provides the first comprehensive experimental evidence and mechanistic dissection of this phenomenon.

The oscillation period of RelB (1.5–2.0 h) is strikingly similar to that of RelA in the canonical pathway (Ashall et al. 2009; Nelson et al. 2004; Tay et al. 2010), suggesting that shared negative feedback mechanisms, particularly IκBα‐mediated nuclear export of NF‐κB subunits, operate across both pathways. Consistent with this, we found that CRM1‐mediated nuclear export and ongoing protein synthesis are both required for RelB oscillations, as previously shown for RelA (Nelson et al. 2004; Sung et al. 2009). However, a key pathway‐specific requirement also emerged. In *Nfkbia* ^*NES/NES*^ MEFs lacking a functional IκBα NES, the proportion of oscillating cells was dramatically reduced, with most cells displaying prolonged activation instead. This suggests that nuclear export of RelB‐IκBα complexes via the IκBα NES is essential for resetting the system and enabling sustained oscillations, a requirement more stringent than that reported for RelA (Hoffmann et al. 2002; Nelson et al. 2004).

A striking contrast with the canonical pathway is the marked population heterogeneity in RelB responses. Whereas canonical pathway stimulation can synchronize oscillations in nearly all cells under appropriate conditions (Ashall et al. 2009; Kellogg and Tay 2015; Zambrano et al. 2016), only approximately 40% of cells oscillated even at saturating LTβR mAb concentrations. Our experiments with *Nfkb2* ^*−/−*^ MEFs provide mechanistic insight into this heterogeneity. In *Nfkb2* knockdown cells, approximately 80% of cells displayed spontaneous oscillatory behavior, and *Nfkb2* ^*−/−*^ cells also showed a marked increase in oscillating cells (50%–60%) compared with LTβR‐stimulated wild‐type cells (~40%), demonstrating that p100 controls oscillation probability through cytoplasmic sequestration of RelB. Since LTβR stimulation induces only partial p100 processing, the extent of RelB release likely varies from cell to cell. Cells with low p100 expression or efficient p100 processing may release sufficient RelB to engage the oscillatory machinery, while others do not. This likely accounts for why only approximately 40% of cells exhibit oscillatory behavior. This post‐receptor threshold mechanism is distinct from the canonical pathway, where response thresholds are set primarily at the level of IKK activation (Kellogg and Tay 2015; Tay et al. 2010). Such population heterogeneity may confer biological advantages, allowing tissues to maintain diverse transcriptional states while remaining capable of mounting oscillatory responses upon sufficiently strong noncanonical stimulation.

Our gene expression data reveal that disruption of RelB oscillatory dynamics in *Nfkbia* ^*NES/NES*^ MEFs is associated with markedly reduced induction of *Spib*, *Traf1*, *Cxcl11*, and *Mmp9* following LTβR stimulation. However, an important caveat applies. *Nfkbia* ^*NES/NES*^ cells also have impaired canonical NF‐κB signaling and reduced basal RelB and p100 expression (Wuerzberger‐Davis et al. 2011), and LTβR stimulation may weakly activate the canonical pathway (Basak et al. 2007). Therefore, whether the impaired gene induction reflects specifically disrupted RelB oscillations or the broader consequences of IκBα nuclear accumulation remains to be determined. Future experiments using more selective perturbations, such as NIK/IKKα inhibition, will be required to establish the specific contribution of RelB oscillations to target gene expression.

We note two technical limitations of this study. In *Ikbkg* ^*−/−*^ and *Nfkbia* ^*NES/NES*^ MEFs, RelB‐Venus was introduced via lentiviral vector rather than the endogenous promoter, meaning that canonical pathway‐dependent regulation of RelB expression levels was not fully recapitulated. Additionally, SV40‐mediated immortalization may induce basal NF‐κB activation, potentially influencing the observed dynamics. Future studies using knock‐in approaches or primary cells will help address these limitations.

Several important questions remain open. Whether RelB oscillations can be entrained by pulsatile stimulation, as demonstrated for RelA (Ashall et al. 2009; Kellogg and Tay 2015; Zambrano et al. 2016), and whether oscillatory versus non‐oscillatory dynamics differentially impact lymphoid organogenesis or B cell maturation in vivo, are key directions for future investigation. In summary, our findings reveal that RelB oscillations share core mechanisms with the canonical pathway while exhibiting unique features, including pronounced population heterogeneity and p100‐dependent threshold control, and provide a foundation for understanding how oscillatory dynamics regulate immune gene expression through the noncanonical NF‐κB pathway.

## 4 Experimental Procedures

### 4.1 Cell Culture and Drug Treatment

Mouse embryonic fibroblasts derived from 2 × FLAG‐tagged RelB‐Venus knock‐in mice (Seki et al. 2015) and *Nfkbia* ^NES/NES^ mice (Wuerzberger‐Davis et al. 2011) were immortalized using lentiviral transduction of the SV40 large T antigen. *Ikbkg* ^−/−^ MEFs were kindly provided by Marc Schmidt‐Supprian (Technical University of Munich). These MEFs were maintained in low glucose Dulbecco's modified Eagle's medium (DMEM) (Wako, 041‐29775) supplemented with 10% fetal bovine serum (FBS). Plat‐E cells (kindly provided by T. Kitamura) and HEK293T cells were maintained in high glucose DMEM (Wako, 043‐30085) supplemented with 10% FBS. For LTβR stimulation, a mixture of anti‐LTβR monoclonal antibodies clone 4H8 WH2 (Adipogen, AG‐20B‐0008‐C100) and clone 3C8 (eBioscience, 16‐5671‐82) at a 10:1 ratio was used at a final concentration of 1 μg/mL. LMB (Sigma, L2913) was added to a final concentration of 1 ng/mL. CHX (Nacalai Tesque, 06741‐62) was used at a final concentration of 2.5 μg/mL. Recombinant mouse TNF (PeproTech, 400‐14) was used at a final concentration of 10 ng/mL.

### 4.2 Plasmid Construction

The lentiviral vector pPS‐RelB‐Venus‐IRES‐puro was designed to express RelB‐Venus fusion protein under the control of the native human *RELB* promoter. *RelB‐Venus* cDNA was amplified by PCR from genomic DNA of MEFs. A 1000 bp region upstream of the human *RELB* gene was cloned by PCR from HEK293T cells. An IRES‐puro fragment was obtained from pMXs‐IRES‐puro (kindly provided by T. Kitamura). The human *RELB* promoter, *RelB‐Venus* cDNA, and IRES‐puro fragments were sequentially inserted into the pPS‐EF1‐LCS‐T2A‐RFP vector (System Biosciences, LF520A‐1) to generate pPS‐RelB‐Venus‐IRES‐puro. sgRNAs targeting *Nfkb2* and *Nfkbia* were designed and selected using the Optimized CRISPR Design tool (http://crispr.mit.edu/). Two complementary oligonucleotides were annealed, phosphorylated, and inserted into AflII‐digested gRNA cloning vector (Addgene, #41824). The sgRNA sequences are listed in Table S1.

### 4.3 Western Blotting

Cells were lysed with Laemmli SDS sample buffer (2% (v/v) SDS, 10% (v/v) glycerol, 5% (v/v) 2‐mercaptoethanol, 62.5 mM Tris–HCl (pH 6.8), and 0.002% (v/v) bromophenol blue). Samples were boiled at 95°C for 10 min. After centrifugation, the cell lysates were subjected to SDS‐PAGE and then transferred onto polyvinylidene difluoride (PVDF) membranes (Millipore, IPVH00010). The membranes were analyzed by immunoblotting with the antibodies listed in Table S2 and developed with Immobilon Western Chemiluminescent HRP substrate (Millipore, WBKL0500). The signals were detected and analyzed using a ChemiDoc imaging system (Bio‐Rad).

### 4.4 Cell Fractionation and Immunoprecipitation

Cells (1 × 10^7^ cells per 15 cm dish) were washed once with ice‐cold PBS and gently scraped using a rubber policeman. The cells were collected in a 50 mL tube and centrifuged at 1500 rpm for 5 min at 4°C. The cell pellet was resuspended in 2 mL PBS, transferred to a 2 mL tube, and centrifuged at 1000*g* for 5 min at 4°C.

For cytoplasmic extraction, the cell pellet was resuspended in 1950 μL hypotonic buffer (10 mM HEPES pH 7.9, 10 mM KCl, 1.5 mM MgCl_2_, and protease inhibitor cocktail) by gentle pipetting and incubated on ice for 30 min. NP‐40 was then added to a final concentration of 0.25%, and the suspension was mixed gently by pipetting. After centrifugation at 1000*g* for 15 min at 4°C, the supernatant was collected as the cytoplasmic fraction. NaCl was added to the cytoplasmic fraction to a final concentration of 100 mM.

For nuclear extraction, the pellet was resuspended with 2 mL hypotonic buffer and centrifuged at 1000*g* for 15 min at 4°C. The pellet was then resuspended in 500 μL IP buffer (1% (v/v) NP‐40, 10 mM Tris–HCl pH 7.5, 100 mM NaCl, 5 mM MgCl_2_, 10% (v/v) glycerol, benzonase (1:2000, Sigma‐Aldrich, E1014), and protease inhibitor cocktail). The suspension was rotated for 2 h at 4°C and centrifuged at 1000*g* for 15 min at 4°C. The supernatant was collected as the nuclear fraction.

For immunoprecipitation, both the cytoplasmic and the nuclear fraction were first pre‐cleared by incubation with 30 μL of Protein G Sepharose 4 Fast Flow (Cytiva, 17‐0618‐02) for 1 h at 4°C with rotation. After centrifugation at 3000 rpm for 2 min at 4°C, the supernatant was incubated with 2 μg of anti‐FLAG M2 antibody (Sigma‐Aldrich, F1804) or mouse normal IgG (Santa Cruz Biotechnology, sc‐2025) overnight at 4°C with rotation. Subsequently, 30 μL of Protein G Sepharose 4 Fast Flow was added, and the mixture was rotated for 1 h at 4°C. The beads were collected by centrifugation at 3000 rpm for 2 min at 4°C and washed three times with IP buffer containing 0.2% (v/v) NP‐40 by gentle inversion. The immunoprecipitates were eluted with 80 μL of 1× sample buffer, boiled at 95°C for 10 min, and subjected to immunoblotting analysis.

### 4.5 siRNA Transfection

For siRNA‐mediated knockdown experiments, cells (4 × 10^5^ cells per well) were transfected with siRNAs using Lipofectamine RNAiMAX (Thermo Fisher Scientific, 13,778,150) according to the manufacturer's protocol. Reverse transfection was initially performed, followed by a second (forward) transfection 17 h later. For both transfection steps, siRNAs and RNAiMAX were diluted in Opti‐MEM (Thermo Fisher Scientific, 31,985,070). At 17 h after the second transfection, culture media were replaced with fresh media, and cells were used for subsequent experiments. The target sequences are listed in Table S3.

### 4.6 Generation of Knockout Cells

The gRNA cloning vector and pCAG‐*hCas9* (Addgene, 51,142) were co‐transfected into cells using Turbofect transfection reagent (Thermo Fisher Scientific, R0531) according to the manufacturer's instructions. At 48 h post‐transfection, cells were seeded onto 96‐well plates by limiting dilution to obtain single‐cell‐derived clones. Knockout of IκBα and p100/p52 in individual clones was confirmed by Western blotting analysis.

### 4.7 Time‐Lapse Microscopy

MEFs were stained with 100 ng/mL Hoechst 33342 (Wako, 080‐09981) in assay medium for 2 h before imaging. Confocal microscopy was performed using an Olympus FV1000D inverted microscope (IX81) equipped with a 20× objective. Cells were cultured in 8‐well chambered coverglass (Nunc, 155,409) maintained in a humidified CO_2_ incubator at 37°C with 5% CO_2_. Hoechst 33342 fluorescence was excited using a 405 nm semiconductor laser and detected through a 435–455 nm bandpass filter. Venus fluorescence was excited using a 515 nm argon ion laser, and emitted light was collected through a 535–565 nm bandpass filter with a 560 nm dichroic mirror. Image acquisition and analysis were performed using FV10‐ASW software (Olympus). Following the addition of stimulation medium, the chambers were sealed and imaged every 5 to 10 min in both Venus and Hoechst 33342 fluorescence channels for up to 15 h.

### 4.8 Cell Tracking and Fluorescence Quantification

Cell images were captured using an Olympus FV1000D microscope and analyzed using NIS‐Elements Advanced Research software (Nikon) to identify nuclei, determine cell boundaries, and quantify fluorescence intensities. In each time‐lapse field, cells that moved out of view or focus, died, overlapped with other cells, or divided during the time course were excluded from analysis.

For nuclear segmentation, nuclei were semi‐automatically identified from Hoechst 33342 images, and mean nuclear RelB‐Venus intensities were extracted. When automatic segmentation failed, nuclear boundaries were marked manually. For whole‐cell quantification, cell boundaries were manually traced from the initial frame of the time‐lapse series, and mean total cellular RelB‐Venus intensity was measured for each cell. Background intensity was measured in manually selected regions without cells and subtracted from both nuclear and total cellular intensities. The nuclear‐to‐total fluorescence ratio (N/T ratio) was calculated by dividing total nuclear intensity (mean nuclear intensity × nuclear area) by total cellular intensity (mean cellular intensity × cellular area).

### 4.9 Fourier Analysis

Each time course profile was analyzed by Fast Fourier Transform (FFT) to identify periodic components. First, each time course *y*(*t*) (nuclear‐to‐total ratio of RelB‐Venus) was adjusted by baseline subtraction and normalization:

$$y_{\text{signal}}\left(t\right)=\frac{y\left(t\right)-\min y\;\left(t\right)}{\max y\left(t\right)-\min y\left(t\right)}$$

The time series was then zero‐padded to extend toward negative *t* values. The $y_{\text{signal}}\left(t\right)$ was further processed using a Hamming window to produce $y_{\text{hamming}}\left(t\right)$. FFT was then applied to $y_{\text{hamming}}\left(t\right)$, and the corresponding power spectrum (periodogram) was generated. A time course was classified as oscillating if the periodogram showed either a single global maximum or a sharp local maximum at a period of less than 5 h. The validity of the FFT analysis was confirmed by verifying that inverse FFT reconstruction accurately restored the original RelB oscillatory profiles.

### 4.10 Peak Detection and Classification of RelB Dynamics

To objectively classify RelB dynamics, peak detection analysis was performed on the N/T ratio time courses. First, the N/T ratio data were smoothed using a Savitzky–Golay filter (polynomial order 2, frame length 7) in MATLAB (MathWorks) to reduce noise while preserving peak features. To determine an appropriate threshold for peak detection, we analyzed unstimulated control cells to establish a MinPeakProminence value that would not detect spurious peaks in the absence of stimulation. Through systematic testing, we found that a MinPeakProminence threshold of 0.09 effectively excluded false peak detection in unstimulated cells. This threshold was then applied to all subsequent peak detection analyses using the MATLAB findpeaks function with MinPeakProminence set to 0.09. Based on the detected peaks and N/T ratio dynamics, RelB behavior was classified into four categories. Cells with no detected peaks were classified as “non‐responding.” Cells showing a single detected peak were classified as “transient activation.” Cells with no detected peaks but exhibiting an N/T ratio increase of 0.09 or greater at the end of imaging compared to the initial time point were classified as “prolonged activation.” Finally, cells with two or more detected peaks were classified as “oscillating.”

### 4.11 Quantitative Real‐Time PCR Analysis

Total RNA was extracted from MEFs using Sepasol‐RNA I SuperG (Nacalai Tesque, 09379‐55), following the manufacturers' protocols. Complementary DNA (cDNA) was synthesized using the ReverTra Ace qPCR RT Kit (Toyobo, FSQ‐101). Quantitative PCR was conducted on a QuantStudio 3 Real‐Time PCR System (Thermo Fisher Scientific) using SYBR Green chemistry. Gene expression levels were normalized to the endogenous control *Hprt* and analyzed using QuantStudio Design & Analysis Software v2.6. The sequences of the primers used are listed in Table S4.

### 4.12 Statistical Analysis

Statistical analysis was performed by two‐tailed unpaired Student's *t*‐test, two‐way ANOVA with Tukey's multiple comparison test, or one‐way ANOVA with Tukey's multiple comparison test using GraphPad Prism 11. *p*‐value < 0.05 was considered significant.

## Author Contributions

**Taishin Akiyama:** writing – review and editing, supervision. **Shelly Davis:** writing – review and editing, methodology, resources. **Hiroyasu Nakano:** writing – review and editing, supervision. **Katsuhide Okunishi:** writing – review and editing, supervision, writing – original draft. **Takao Seki:** writing – review and editing, conceptualization, methodology, software, data curation, investigation, visualization, project administration, writing – original draft, funding acquisition, validation, formal analysis. **Jun‐ichiro Inoue:** writing – review and editing, supervision, funding acquisition, conceptualization. **Shigeki Miyamoto:** writing – review and editing, methodology, resources, funding acquisition.

## Funding

This work was supported by a Grant‐in‐Aid for Scientific Research on Scientific Research (B) (18390409, 20390393, and 26290036 to JI); a Grant‐in‐Aid for Scientific Research on Innovative Areas (22117002 and 16H06575 to JI); a Grant‐in‐Aid for Early‐Career Scientists (20K16151 to TS); a grant‐in‐aid for the Genome Network Project (1005679 to JI); contract research funding from the Japan Initiative for Global Research Network on Infectious Diseases (925190 to JI) from the Ministry of Education, Culture, Sports, Science, and Technology of Japan; and NIH R01CA246321 (to SM).

## Conflicts of Interest

The authors declare no conflicts of interest.

## Supporting information


**Figure S1:** RelB oscillations are induced by multiple non‐canonical NF‐κB pathway stimuli.
Experiments in (A–C) were performed using primary MEFs (pMEFs) derived from RelB‐Venus knock‐in mice. Experiments in (D–G) were performed using immortalized MEFs (iMEFs) established from RelB‐Venus knock‐in mice.
(A) Single‐cell N/T ratio traces for each response category in pMEFs stimulated with anti‐LTβR mAb (1 μg/mL). Five representative cells per category are shown.
(B) Fraction of pMEFs displaying each response pattern following anti‐LTβR mAb stimulation (1 μg/mL).
(C) Distribution of oscillation periods in oscillating pMEFs determined by FFT analysis.
(D) Fraction of iMEFs (clones #2 and #3) displaying each response pattern at the indicated anti‐LTβR mAb concentrations.
(E) Single‐cell N/T ratio traces for oscillating iMEFs (clone #1) stimulated with TWEAK. Five representative cells are shown.
(F) Fraction of iMEFs (clone #1) displaying each response pattern at the indicated TWEAK concentrations.
(G) Distribution of oscillation periods in oscillating iMEFs (clone #1) stimulated with TWEAK, determined by FFT analysis.
All experiments are representative of at least three independent experiments.
**Figure S2:** NIK is required for RelB oscillations in the non‐canonical NF‐κB pathway.
Experiments in (A–C) were performed using *Ikbkg* ^*⁻/⁻*^ iMEFs transduced with lentiviral RelB‐Venus vector. Experiments (D–F) were performed using RelB‐Venus knock‐in iMEFs.
(A) Western blot analysis of *Ikbkg* ^*+/+*^ and *Ikbkg* ^*−/−*^ iMEFs. Upper panels: IκBα degradation in response to TNFα (10 ng/mL) at the indicated time points. Lower panels: p100 processing following anti‐LTβR mAb stimulation (1 μg/mL) at the indicated time points.
(B) Single‐cell N/T ratio traces for oscillating *Ikbkg* ^*+/+*^ and *Ikbkg* ^*−/−*^ iMEFs stimulated with anti‐LTβR mAb (1 μg/mL). Five representative cells per genotype are shown.
(C) Fraction of *Ikbkg* ^*+/+*^ and *Ikbkg* ^*−/−*^ iMEFs displaying each response pattern following anti‐LTβR mAb stimulation (1 μg/mL).
(D) Western blot analysis of NIK protein levels in iMEFs transfected with control or *Map3k14* siRNA (#1 and #2), with or without MG132 treatment (10 μM, 4 h).
(E) Western blot analysis of iMEFs transfected with control or *Map3k14* siRNA. Upper panels: IκBα degradation in response to TNFα (10 ng/mL). Lower panels: p100 processing following anti‐LTβR mAb stimulation (1 μg/mL).
(F) Fraction of iMEFs transfected with control or *Map3k14* siRNA displaying each response pattern following anti‐LTβR mAb stimulation (1 μg/mL).
Statistical significance was determined by two‐tailed unpaired Student's *t*‐test (C) or one‐way ANOVA with Tukey's multiple comparisons test (F): **p < 0.01; ***p < 0.001; ****p < 0.0001; ns, not significant. All experiments are representative of at least two independent experiments.
**Figure S3:** p100 controls RelB oscillation threshold by cytoplasmic sequestration.
All experiments were performed using *Nfkb2⁻/⁻* iMEFs generated from RelB‐Venus knock‐in mice by CRISPR‐Cas9 genome editing.
(A) CRISPR‐Cas9 targeting strategy for generating *Nfkb2* ^*−/−*^ iMEFs. The guide RNA sequence targeting exon 5 of the *Nfkb2* locus is shown in red, with the PAM sequence indicated.
(B) Western blot analysis of p100, p52, RelB, IκBα, and Tubulin in *Nfkb2* ^*+/+*^, *Nfkb2* ^*−/−*^ #1, and *Nfkb2* ^*−/−*^ #2 iMEFs without LTβR stimulation.
(C) Single‐cell N/T ratio traces in *Nfkb2* ^*−/−*^ #1 (left), *Nfkb2* ^*−/−*^ #2 (middle), and *Nfkb2* ^*+/+*^ (right) iMEFs without stimulation (no stimulation). Five representative cells per genotype are shown.
(D) Fraction of *Nfkb2* ^*+/+*^ and *Nfkb2* ^*−/−*^ iMEFs displaying each response pattern without LTβR stimulation.
(E) Distribution of oscillation periods in spontaneously oscillating *Nfkb2* ^*−/−*^ #1 (left) and *Nfkb2* ^*−/−*^ #2 (right) iMEFs determined by FFT analysis.
Statistical significance was determined by one‐way ANOVA with Tukey's multiple comparisons test (D): **p < 0.01; ***p < 0.001; ****p < 0.0001. All experiments are representative of at least three independent experiments.
**Figure S4:** IκBα contributes to but is not required for RelB oscillations.
All experiments were performed using *Nfkbia⁻/⁻* iMEFs generated from RelB‐Venus knock‐in mice by CRISPR‐Cas9 genome editing.
(A) CRISPR‐Cas9 targeting strategy for generating *Nfkbia* ^*−/−*^ iMEFs. Two guide RNA sequences targeting exon 1 of the *Nfkbia* locus are shown in red, with the ATG start codon and PAM sequences indicated.
(B) Western blot analysis of IκBα, p100, p52, RelB, and Tubulin in *Nfkbia* ^*+/+*^, *Nfkbia* ^*−/−*^ #1, and *Nfkbia* ^*−/−*^ #2 iMEFs.
(C) Fraction of *Nfkbia* ^*+/+*^, *Nfkbia* ^*−/−*^ #1, and *Nfkbia* ^*−/−*^ #2 iMEFs displaying each response pattern following anti‐LTβR mAb stimulation (1 μg/mL).
(D) Single‐cell N/T ratio traces in *Nfkbia* ^*+/+*^ (left), *Nfkbia* ^*−/−*^ #1 (middle), and *Nfkbia* ^*−/−*^ #2 (right) iMEFs following anti‐LTβR mAb stimulation (1 μg/mL). Five representative cells per genotype are shown.
(E) Distribution of oscillation periods in oscillating *Nfkbia* ^*+/+*^ (left), *Nfkbia* ^*−/−*^ #1 (middle), and *Nfkbia* ^*−/−*^ #2 (right) iMEFs determined by FFT analysis.
Statistical significance was determined by one‐way ANOVA with Tukey's multiple comparisons test (C): ns, not significant. All experiments are representative of at least three independent experiments.
**Figure S5:** RelB forms distinct protein complexes in *Nfkb2* ^*+/+*^ and *Nfkb2* ^*−/−*^ iMEFs.
All experiments were performed using *Nfkb2⁻/⁻* iMEFs generated from RelB‐Venus knock‐in mice by CRISPR‐Cas9 genome editing.
(A) Co‐immunoprecipitation of RelB‐Venus‐2 × FLAG from the cytoplasmic fraction of *Nfkb2* ^*+/+*^, *Nfkb2* ^*−/−*^ #1, and *Nfkb2* ^*−/−*^ #2 iMEFs without LTβR stimulation. IgG immunoprecipitation of *Nfkb2* ^*+/+*^ cells serves as a negative control. FLAG immunoprecipitates and input fractions were analyzed by western blot for IκBα, p105, p50, p100, p52, and RelB. Tubulin and HDAC2 serve as cytoplasmic and nuclear fraction markers, respectively.
(B) Co‐immunoprecipitation of RelB‐Venus‐2 × FLAG from the nuclear fraction of *Nfkb2* ^*+/+*^, *Nfkb2* ^*−/−*^ #1, and *Nfkb2* ^*−/−*^ #2 iMEFs. IgG immunoprecipitation of *Nfkb2* ^*+/+*^ cells serves as a negative control. Samples were analyzed as in (A).
All experiments are representative of at least two independent experiments.
**Table S1:** sgRNA sequences.
**Table S2:** Antibodies for Western blotting.
**Table S3:** siRNA sequences.
**Table S4:** Primer sequences.

## Data Availability

The data that support the findings of this study are available from the corresponding author upon reasonable request. The authors intend to deposit the data in the Toho University institutional repository upon acceptance of this manuscript.

## References

[gtc70135-bib-0001] Ashall, L. , C. A. Horton , D. E. Nelson , et al. 2009. “Pulsatile Stimulation Determines Timing and Specificity of NF‐kappaB‐Dependent Transcription.” Science 324, no. 5924: 242–246. 19359585 10.1126/science.1164860 PMC2785900

[gtc70135-bib-0002] Basak, S. , H. Kim , J. D. Kearns , et al. 2007. “A Fourth IkappaB Protein Within the NF‐kappaB Signaling Module.” Cell 128, no. 2: 369–381. 17254973 10.1016/j.cell.2006.12.033 PMC1831796

[gtc70135-bib-0003] Busino, L. , S. E. Millman , L. Scotto , et al. 2012. “Fbxw7α‐ and GSK3‐Mediated Degradation of p100 Is a Pro‐Survival Mechanism in Multiple Myeloma.” Nature Cell Biology 14, no. 4: 375–385. 22388891 10.1038/ncb2463 PMC3339029

[gtc70135-bib-0004] Hayden, M. S. , and S. Ghosh . 2012. “NF‐κB, the First Quarter‐Century: Remarkable Progress and Outstanding Questions.” Genes & Development 26, no. 3: 203–234. 22302935 10.1101/gad.183434.111 PMC3278889

[gtc70135-bib-0005] Hoffmann, A. , A. Levchenko , M. L. Scott , and D. Baltimore . 2002. “The IkappaB‐NF‐kappaB Signaling Module: Temporal Control and Selective Gene Activation.” Science 298, no. 5596: 1241–1245. 12424381 10.1126/science.1071914

[gtc70135-bib-0006] Kellogg, R. A. , and S. Tay . 2015. “Noise Facilitates Transcriptional Control Under Dynamic Inputs.” Cell 160, no. 3: 381–392. 25635454 10.1016/j.cell.2015.01.013

[gtc70135-bib-0007] Levine, J. H. , Y. Lin , and M. B. Elowitz . 2013. “Functional Roles of Pulsing in Genetic Circuits.” Science 342, no. 6163: 1193–1200. 24311681 10.1126/science.1239999 PMC4100686

[gtc70135-bib-0008] Nelson, D. E. , A. E. C. Ihekwaba , M. Elliott , et al. 2004. “Oscillations in NF‐kappaB Signaling Control the Dynamics of Gene Expression.” Science 306, no. 5696: 704–708. 15499023 10.1126/science.1099962

[gtc70135-bib-0009] Purvis, J. E. , and G. Lahav . 2013. “Encoding and Decoding Cellular Information Through Signaling Dynamics.” Cell 152, no. 5: 945–956. 23452846 10.1016/j.cell.2013.02.005 PMC3707615

[gtc70135-bib-0010] Seki, T. , M. Yamamoto , Y. Taguchi , et al. 2015. “Visualization of RelB Expression and Activation at the Single‐Cell Level During Dendritic Cell Maturation in Relb‐Venus Knock‐In Mice.” Journal of Biochemistry 158, no. 6: 485–495. 26115685 10.1093/jb/mvv064

[gtc70135-bib-0011] Senftleben, U. , Y. Cao , G. Xiao , et al. 2001. “Activation by IKKalpha of a Second, Evolutionary Conserved, NF‐Kappa B Signaling Pathway.” Science 293, no. 5534: 1495–1499. 11520989 10.1126/science.1062677

[gtc70135-bib-0012] Sun, S.‐C. 2017. “The Non‐Canonical NF‐κB Pathway in Immunity and Inflammation.” Nature Reviews Immunology 17, no. 9: 545–558. 10.1038/nri.2017.52 PMC5753586 28580957

[gtc70135-bib-0013] Sung, M.‐H. , L. Salvatore , R. De Lorenzi , et al. 2009. “Sustained Oscillations of NF‐kappaB Produce Distinct Genome Scanning and Gene Expression Profiles.” PLoS One 4, no. 9: e7163. 19787057 10.1371/journal.pone.0007163 PMC2747007

[gtc70135-bib-0014] Tay, S. , J. J. Hughey , T. K. Lee , T. Lipniacki , S. R. Quake , and M. W. Covert . 2010. “Single‐Cell NF‐kappaB Dynamics Reveal Digital Activation and Analogue Information Processing.” Nature 466, no. 7303: 267–271. 20581820 10.1038/nature09145 PMC3105528

[gtc70135-bib-0015] Umegaki, T. , N. Hatanaka , and T. Suzuki . 2024. “Mathematical Structure of RelB Dynamics in the NF‐κB Non‐Canonical Pathway.” Mathematical and Computational Applications 29, no. 4: 62.

[gtc70135-bib-0016] Werner, S. L. , D. Barken , and A. Hoffmann . 2005. “Stimulus Specificity of Gene Expression Programs Determined by Temporal Control of IKK Activity.” Science 309, no. 5742: 1857–1861. 16166517 10.1126/science.1113319

[gtc70135-bib-0017] Wuerzberger‐Davis, S. M. , Y. Chen , D. T. Yang , et al. 2011. “Nuclear Export of the NF‐κB Inhibitor IκBα Is Required for Proper B Cell and Secondary Lymphoid Tissue Formation.” Immunity 34, no. 2: 188–200. 21333553 10.1016/j.immuni.2011.01.014 PMC3111750

[gtc70135-bib-0018] Xiao, G. , E. W. Harhaj , and S. C. Sun . 2001. “NF‐kappaB‐Inducing Kinase Regulates the Processing of NF‐kappaB2 p100.” Molecular Cell 7, no. 2: 401–409. 11239468 10.1016/s1097-2765(01)00187-3

[gtc70135-bib-0019] Zambrano, S. , I. De Toma , A. Piffer , M. E. Bianchi , and A. Agresti . 2016. “NF‐κB Oscillations Translate Into Functionally Related Patterns of Gene Expression.” eLife 5: e09100. 26765569 10.7554/eLife.09100 PMC4798970

[gtc70135-bib-0020] Zhang, Q. , M. J. Lenardo , and D. Baltimore . 2017. “30 Years of NF‐κB: A Blossoming of Relevance to Human Pathobiology.” Cell 168, no. 1–2: 37–57. 28086098 10.1016/j.cell.2016.12.012 PMC5268070

