# Supplementary material for: The Nuclear Export Signal of IκBα Drives RelB Oscillations in the Noncanonical NF‐κB Pathway
Source: Genes Cells. 2026 Jul 2;31(4):e70135. doi: 10.1111/gtc.70135 (PMC13325521; doi:10.1111/gtc.70135)
Supplement: Supplementary file 1 — Figure S1: RelB oscillations are induced by multiple non‐canonical NF‐κB pathway stimuli. Experiments in (A–C) were performed using primary MEFs (pMEFs) derived from RelB‐Venus knock‐in mice. Experiments in (D–G) were performed using immortalized MEFs (iMEFs) established from RelB‐Venus knock‐in mice. (A) Single‐cell N/T ratio traces for each response category in pMEFs stimulated with anti‐LTβR mAb (1 μg/mL). Five representative cells per category are shown. (B) Fraction of pMEFs displaying each response pattern following anti‐LTβR mAb stimulation (1 μg/mL). (C) Distribution of oscillation periods in oscillating pMEFs determined by FFT analysis. (D) Fraction of iMEFs (clones #2 and #3) displaying each response pattern at the indicated anti‐LTβR mAb concentrations. (E) Single‐cell N/T ratio traces for oscillating iMEFs (clone #1) stimulated with TWEAK. Five representative cells are shown. (F) Fraction of iMEFs (clone #1) displaying each response pattern at the indicated TWEAK concentrations. (G) Distribution of oscillation periods in oscillating iMEFs (clone #1) stimulated with TWEAK, determined by FFT analysis. All experiments are representative of at least three independent experiments. Figure S2: NIK is required for RelB oscillations in the non‐canonical NF‐κB pathway. Experiments in (A–C) were performed using Ikbkg ⁻/⁻ iMEFs transduced with lentiviral RelB‐Venus vector. Experiments (D–F) were performed using RelB‐Venus knock‐in iMEFs. (A) Western blot analysis of Ikbkg +/+ and Ikbkg −/− iMEFs. Upper panels: IκBα degradation in response to TNFα (10 ng/mL) at the indicated time points. Lower panels: p100 processing following anti‐LTβR mAb stimulation (1 μg/mL) at the indicated time points. (B) Single‐cell N/T ratio traces for oscillating Ikbkg +/+ and Ikbkg −/− iMEFs stimulated with anti‐LTβR mAb (1 μg/mL). Five representative cells per genotype are shown. (C) Fraction of Ikbkg +/+ and Ikbkg −/− iMEFs displaying each response pattern following anti‐LTβR mAb stimula [file GTC-31-0-s001.docx]

**Supplementary Information**

Takao Seki^1,2,3^*, Shelly Davis^4^, Shigeki Miyamoto^4^, Taishin Akiyama^2^, Hiroyasu Nakano^5, 6^, Jun-ichiro Inoue^1^, and Katsuhide Okunishi^3^

^1^Division of Cellular and Molecular Biology, Department of Cancer Biology, The Institute of Medical Science, The University of Tokyo, Minato-ku, Tokyo, 108-8639, Japan.

^2^Laboratory for Immune Homeostasis, RIKEN Center for Integrative Medical Sciences, Yokohama, Kanagawa, 230-0045, Japan.

^3^Department of Biochemistry, Faculty of Medicine, Toho University, 5-21-16 Omori-Nishi, Ota-ku, Tokyo 143-8540, Japan.

^4^Department of Oncology, University of Wisconsin Carbone Cancer Center, University of Wisconsin, Madison, Wisconsin 53705.

^5^Unit of Host Defense, Faculty of Medicine, Toho University, 5-21-16 Omori-Nishi, Ota-ku, Tokyo 143-8540, Japan.

^6^Research Administration Organization, Toho University, 5-21-16 Omori-Nishi, Ota-ku, Tokyo 143-8540, Japan.

*Corresponding Author. Email; takao.seki@toho-u.med.co.jp


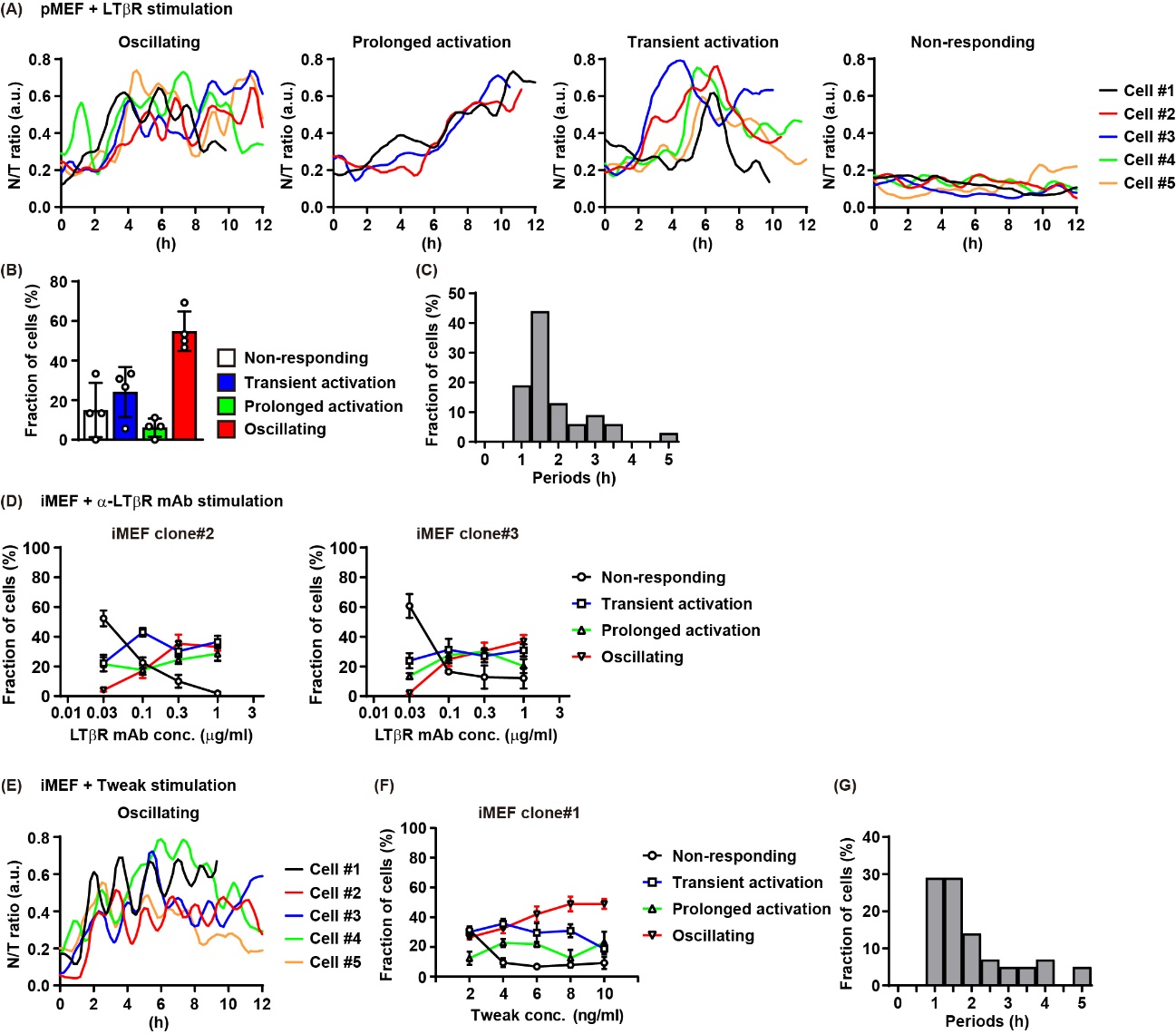


**Figure S1. RelB oscillations are induced by multiple non-canonical NF-κB pathway stimuli**

Experiments in (A–C) were performed using primary MEFs (pMEFs) derived from RelB-Venus knock-in mice. Experiments in (D–G) were performed using immortalized MEFs (iMEFs) established from RelB-Venus knock-in mice.

(A) Single-cell N/T ratio traces for each response category in pMEFs stimulated with anti-LTβR mAb (1 μg/mL). Five representative cells per category are shown.

(B) Fraction of pMEFs displaying each response pattern following anti-LTβR mAb stimulation (1 μg/mL).

(C) Distribution of oscillation periods in oscillating pMEFs determined by FFT analysis.

(D) Fraction of iMEFs (clones #2 and #3) displaying each response pattern at the indicated anti-LTβR mAb concentrations.

(E) Single-cell N/T ratio traces for oscillating iMEFs (clone #1) stimulated with TWEAK. Five representative cells are shown.

(F) Fraction of iMEFs (clone #1) displaying each response pattern at the indicated TWEAK concentrations.

(G) Distribution of oscillation periods in oscillating iMEFs (clone #1) stimulated with TWEAK, determined by FFT analysis.

All experiments are representative of at least three independent experiments.


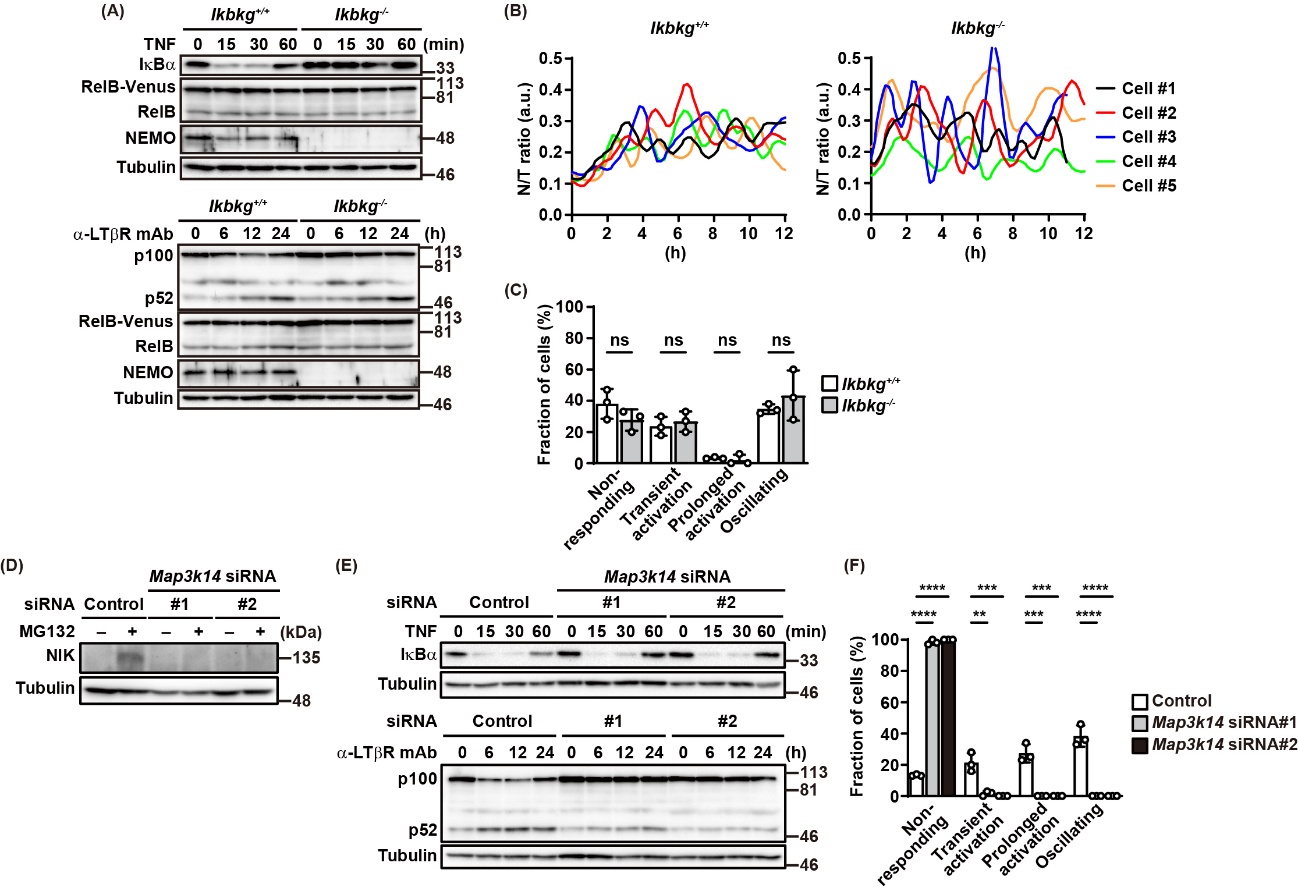


**Figure S2. NIK is required for RelB oscillations in the non-canonical NF-κB pathway**

Experiments in (A–C) were performed using *Ikbkg^⁻/⁻^* iMEFs transduced with lentiviral RelB-Venus vector. Experiments (D–F) were performed using RelB-Venus knock-in iMEFs.

(A) Western blot analysis of *Ikbkg^+/+^* and *Ikbkg^-/-^* iMEFs. Upper panels: IκBα degradation in response to TNFα (10 ng/mL) at the indicated time points. Lower panels: p100 processing following anti-LTβR mAb stimulation (1 μg/mL) at the indicated time points.

(B) Single-cell N/T ratio traces for oscillating *Ikbkg^+/+^* and *Ikbkg^-/-^* iMEFs stimulated with anti-LTβR mAb (1 μg/mL). Five representative cells per genotype are shown.

(C) Fraction of *Ikbkg^+/+^* and *Ikbkg^-/-^* iMEFs displaying each response pattern following anti-LTβR mAb stimulation (1 μg/mL).

(D) Western blot analysis of NIK protein levels in iMEFs transfected with control or *Map3k14* siRNA (#1 and #2), with or without MG132 treatment (10 μM, 4 h).

(E) Western blot analysis of iMEFs transfected with control or *Map3k14* siRNA. Upper panels: IκBα degradation in response to TNFα (10 ng/mL). Lower panels: p100 processing following anti-LTβR mAb stimulation (1 μg/mL).

(F) Fraction of iMEFs transfected with control or *Map3k14* siRNA displaying each response pattern following anti-LTβR mAb stimulation (1 μg/mL).

Statistical significance was determined by two-tailed unpaired Student's *t*-test (C) or one-way ANOVA with Tukey's multiple comparisons test (F): **P < 0.01; ***P < 0.001; ****P < 0.0001; ns, not significant. All experiments are representative of at least two independent experiments.


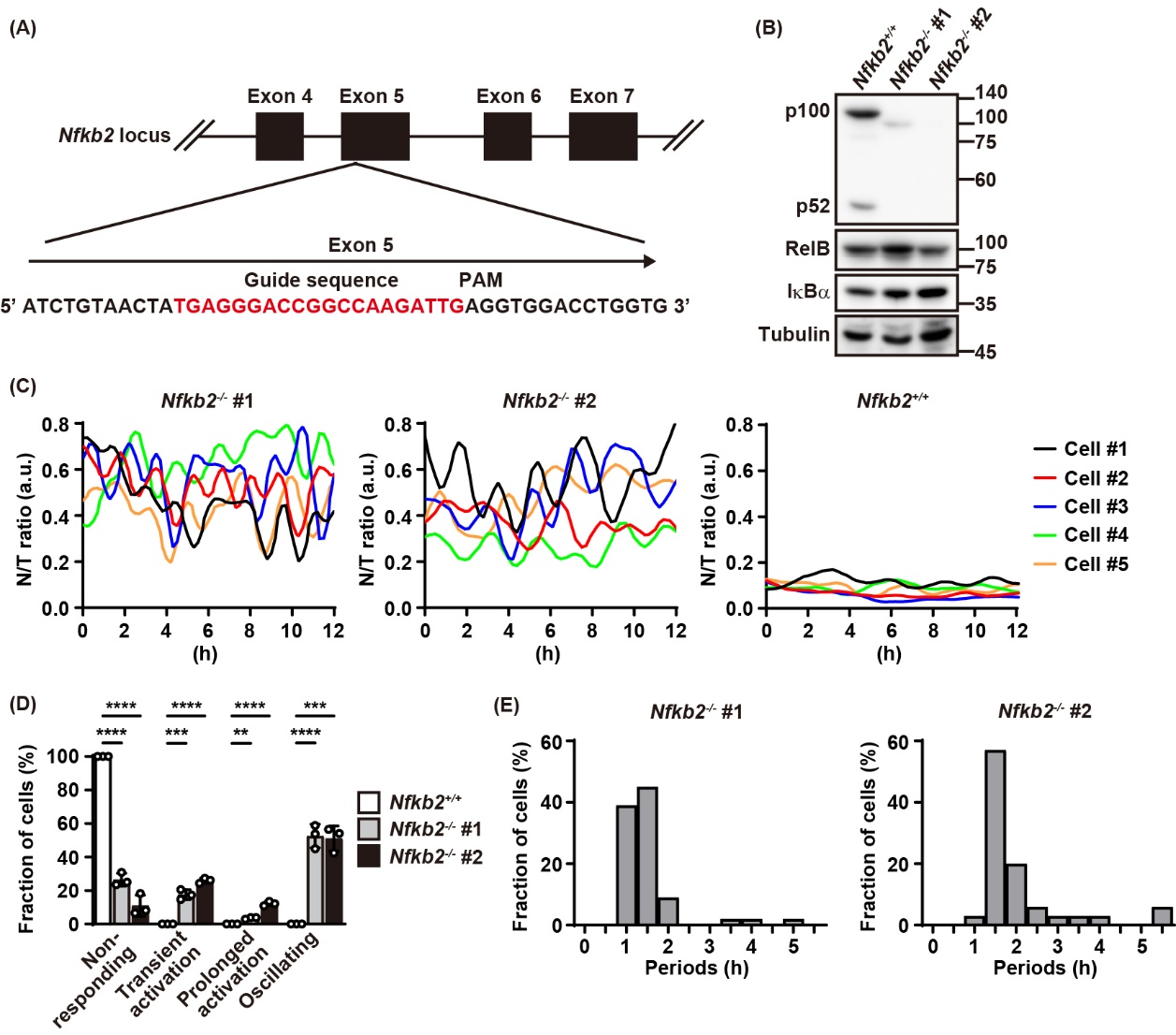


**Figure S3. p100 controls RelB oscillation threshold by cytoplasmic sequestration**

All experiments were performed using *Nfkb2⁻/⁻* iMEFs generated from RelB-Venus knock-in mice by CRISPR-Cas9 genome editing.

(A) CRISPR-Cas9 targeting strategy for generating *Nfkb2^-/-^* iMEFs. The guide RNA sequence targeting exon 5 of the *Nfkb2* locus is shown in red, with the PAM sequence indicated.

(B) Western blot analysis of p100, p52, RelB, IκBα, and Tubulin in *Nfkb2^+/+^*, *Nfkb2^-/-^* #1, and *Nfkb2^-/-^* #2 iMEFs without LTβR stimulation.

(C) Single-cell N/T ratio traces in *Nfkb2^-/-^* #1 (left), *Nfkb2^-/-^* #2 (middle), and *Nfkb2^+/+^* (right) iMEFs without stimulation (no stimulation). Five representative cells per genotype are shown.

(D) Fraction of *Nfkb2^+/+^* and *Nfkb2^-/-^* iMEFs displaying each response pattern without LTβR stimulation.

(E) Distribution of oscillation periods in spontaneously oscillating *Nfkb2^-/-^* #1 (left) and *Nfkb2^-/-^* #2 (right) iMEFs determined by FFT analysis.

Statistical significance was determined by one-way ANOVA with Tukey's multiple comparisons test (D): **p < 0.01; ***p < 0.001; ****p < 0.0001. All experiments are representative of at least three independent experiments.


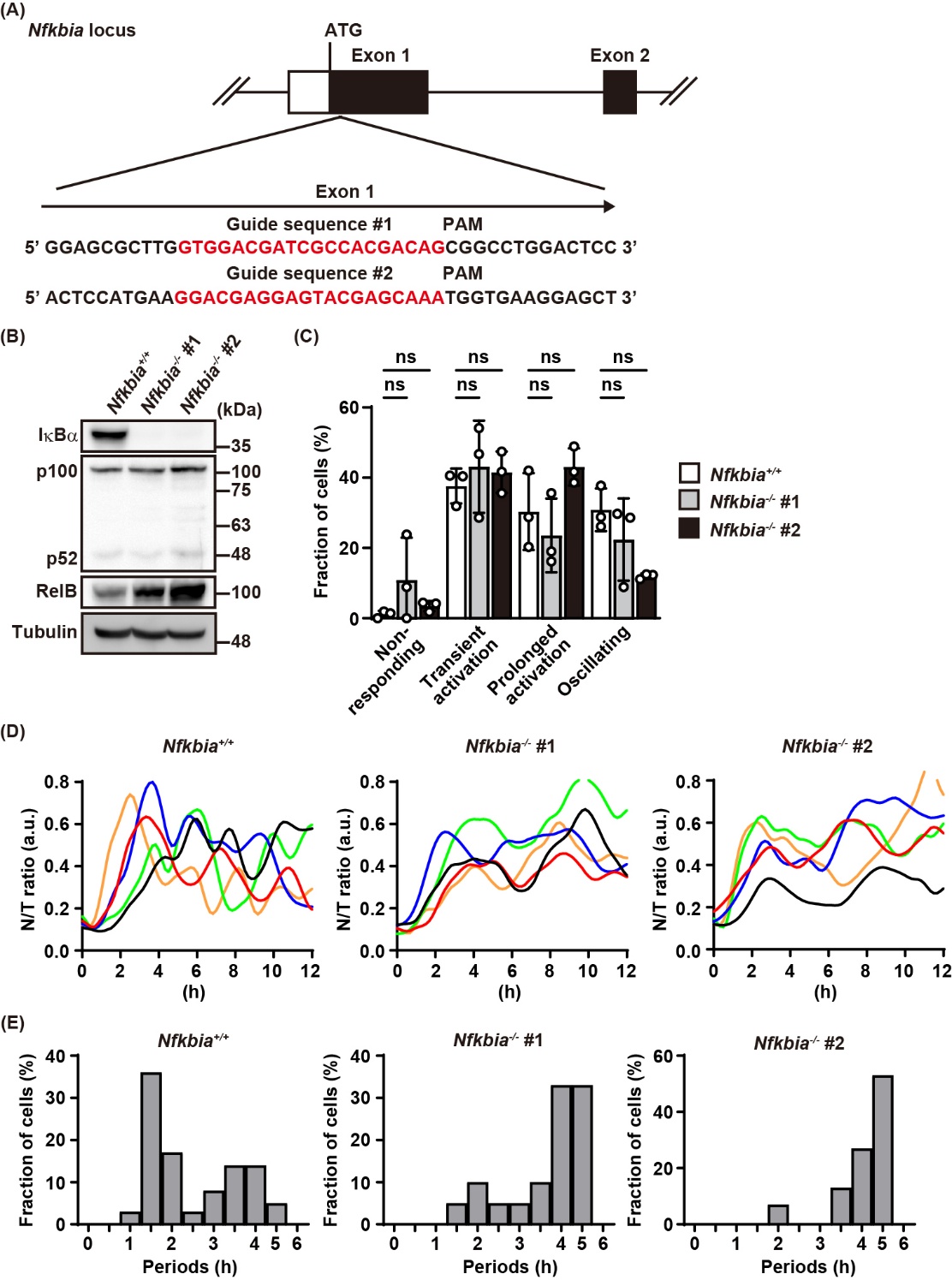


**Figure S4. IκBα contributes to but is not required for RelB oscillations**

All experiments were performed using *Nfkbia⁻/⁻* iMEFs generated from RelB-Venus knock-in mice by CRISPR-Cas9 genome editing.

(A) CRISPR-Cas9 targeting strategy for generating *Nfkbia^-/-^* iMEFs. Two guide RNA sequences targeting exon 1 of the *Nfkbia* locus are shown in red, with the ATG start codon and PAM sequences indicated.

(B) Western blot analysis of IκBα, p100, p52, RelB, and Tubulin in *Nfkbia^+/+^*, *Nfkbia^-/-^* #1, and *Nfkbia^-/-^* #2 iMEFs.

(C) Fraction of *Nfkbia^+/+^*, *Nfkbia^-/-^* #1, and *Nfkbia^-/-^* #2 iMEFs displaying each response pattern following anti-LTβR mAb stimulation (1 μg/mL).

(D) Single-cell N/T ratio traces in *Nfkbia^+/+^* (left), *Nfkbia^-/-^* #1 (middle), and *Nfkbia^-/-^* #2 (right) iMEFs following anti-LTβR mAb stimulation (1 μg/mL). Five representative cells per genotype are shown.

(E) Distribution of oscillation periods in oscillating *Nfkbia^+/+^* (left), *Nfkbia^-/-^* #1 (middle), and *Nfkbia^-/-^* #2 (right) iMEFs determined by FFT analysis.

Statistical significance was determined by one-way ANOVA with Tukey's multiple comparisons test (C): ns, not significant. All experiments are representative of at least three independent experiments.


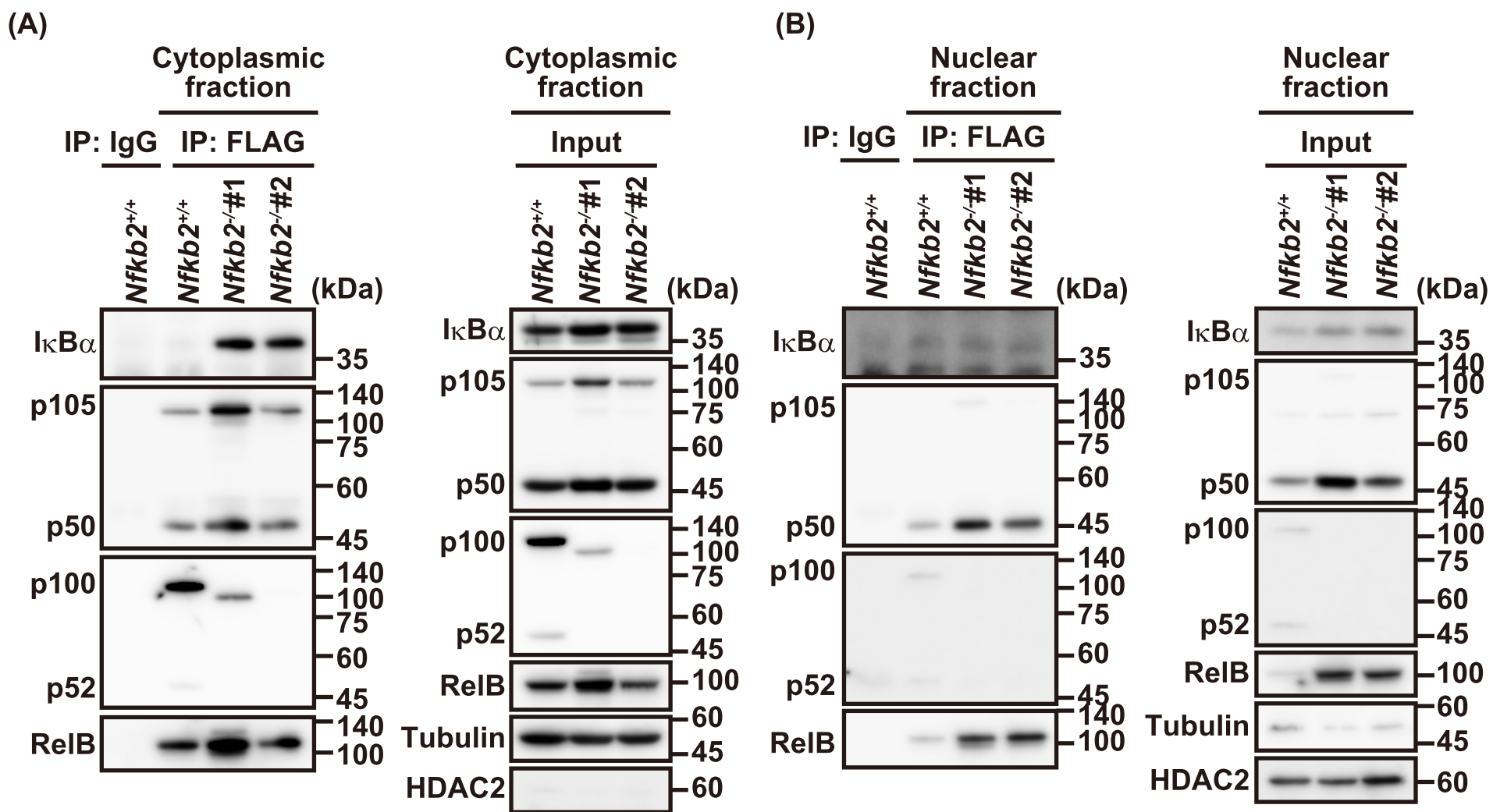


**Figure S5. RelB forms distinct protein complexes in *Nfkb2^+/+^* and *Nfkb2^-/-^* iMEFs**

All experiments were performed using *Nfkb2⁻/⁻* iMEFs generated from RelB-Venus knock-in mice by CRISPR-Cas9 genome editing.

(A) Co-immunoprecipitation of RelB-Venus-2×FLAG from the cytoplasmic fraction of *Nfkb2^+/+^*, *Nfkb2^-/-^* #1, and *Nfkb2^-/-^* #2 iMEFs without LTβR stimulation. IgG immunoprecipitation of *Nfkb2^+/+^* cells serves as a negative control. FLAG immunoprecipitates and input fractions were analyzed by western blot for IκBα, p105, p50, p100, p52, and RelB. Tubulin and HDAC2 serve as cytoplasmic and nuclear fraction markers, respectively.

(B) Co-immunoprecipitation of RelB-Venus-2×FLAG from the nuclear fraction of *Nfkb2^+/+^*, *Nfkb2^-/-^* #1, and *Nfkb2^-/-^* #2 iMEFs. IgG immunoprecipitation of *Nfkb2^+/+^* cells serves as a negative control. Samples were analyzed as in (A).

All experiments are representative of at least two independent experiments.

**Table S1. sgRNA sequences**

| sgRNA | Sequence (5'→3') |
| --- | --- |
| *Nfkb2* Fw | TGAGGGACCGGCCAAGATTGAGG |
| *Nfkb2* Rv | CCTCAATCTTGGCCGGTCCCTCA |
| *Nfkbia* #1 Fw | GTGGACGATCGCCACGACAGCGG |
| *Nfkbia* #1 Rv | CCGCTGTCGTGGCGATCGTCCAC |
| *Nfkbia* #2 Fw | GGACGAGGAGTACGAGCAAATGG |
| *Nfkbia* #2 Rv | CCATTTGCTCGTACTCCTCGTCC |

| Antibody | Company | Catalog No. | Dilution | Incubation |
| --- | --- | --- | --- | --- |
| anti-NIK | Cell Signaling Technology | 4994 | 1:1000 | overnight at 4°C |
| anti-NEMO | Santa Cruz Biotechnology | sc-8330 | 1:1000 | overnight at 4°C |
| anti-IκBα | Cell Signaling Technology | 9242 | 1:2000 | overnight at 4°C |
| anti-p100/p52 | Cell Signaling Technology | 4882 | 1:1000 | overnight at 4°C |
| anti-p105/p50 | Cell Signaling Technology | 13586 | 1:2000 | overnight at 4°C |
| anti-FLAG (M2) | Sigma-Aldrich | A2220-1ML | 1:2000 | overnight at 4°C |
| anti-RelB | Cell Signaling Technology | 4922 | 1:2000 | overnight at 4°C |
| anti-Tubulin | Calbiochem | CP06 | 1:5000 | overnight at 4°C |
| anti-HDAC2 | Cell Signaling Technology | 2540 | 1:2000 | overnight at 4°C |
| anti-mouse IgG-HRP | Cytiva | NA931V | 1:5000 | 1 h at RT |
| anti-Rabbit IgG-HRP | Cytiva | NA934V | 1:5000 | 1 h at RT |

**Table S2. Antibodies for Western blotting**

| siRNA | Sequence (5'→3') |
| --- | --- |
| *Nfkb2* stealth-1 | CCAAGGACAUGACUGCUCAAUUUAA |
| *Nfkb2* stealth-2 | CCAACUCUUACUCGCCUCCUUCUAA |
| *Map3k14* stealth-1 | GGGUCCUGCUUACUGAGAAACUCAA |
| *Map3k14* stealth-2 | GACAGGCUUCCAGUGUGCUGUCAAA |
| Stealth RNAi™ siRNA Negative Control, Med GC | Proprietary sequence |

**Table S3. siRNA sequences**

| Primer | Sequence (5'→3') |
| --- | --- |
| *Spib*_Fwd | AACCACCATGCTTGCTCTGG |
| *Spib*_Rev | TGTAGAGTCAAGGCCCCCAT |
| *Traf1*_Fwd | AGGGTGGTGGAATTACAGCAA |
| *Traf1*_Rev | GCAGTGTAGAAAGCTGGAGAG |
| *Cxcl11*_Fwd | GGCTTCCTTATGTTCAAACAGGG |
| *Cxcl11*_Rev | GCCGTTACTCGGGTAAATTACA |
| *Mmp9*_Fwd | CCCAGCTGGCAGAGGCATAC |
| *Mmp9*_Rev | GGAATCGACCCACGTCTGG |
| *Hprt*_Fwd | AACAAAGTCTGGCCTGTATCCAA |
| *Hprt*_Rev | GCAGTACAGCCCCAAAATGG |

**Table S4. Primer sequences**
